# Evaluation of Laboratory Performance, Associated Factors and Staff Awareness Towards Achieving Turnaround Time in Tertiary Hospitals, Ethiopia

**DOI:** 10.4314/ejhs.v30i5.17

**Published:** 2020-09

**Authors:** Mebrat Gebreyes, Abay Sisay, Dilargachew Tegen, Abushet Asnake, Mistire Wolde

**Affiliations:** 1 Department of Medical Laboratory, Armed Force Comprehensive and Specialized Hospital, Addis Ababa, Ethiopia; 2 Department of Medical Laboratory Sciences, College of Health Sciences, Addis Ababa University, Addis Ababa, Ethiopia

**Keywords:** Turnaround time, performance, Quality indicator, Laboratory, Ethiopia

## Abstract

**Background:**

WHO recommends that each laboratory should establish turnaround time (TAT) to monitor and evaluate performance throughout processes. The status of established TAT was not yet assessed in Ethiopian Armed Force Comprehensive Specialized Hospital. The aim of this study was to evaluate the laboratory performance and associated factors towards achieving TAT in clinical chemistry and hematology tests at Armed Force Comprehensive Specialized Hospital, Addis Ababa, Ethiopia.

**Methods:**

Hospital-based cross-sectional study was conducted from April 2019 to June 2019. Standardized questionnaire was designed to collected data on awareness of laboratory staffs about TAT. The data was entered, cleaned and analyzed using SPSS version 24.0 Software. Logistic regression analysis was done to find out statistically significant association and strength of association between dependent and independent variables at pvalue <0.05.

**Result:**

A total of 422 test results were systematically selected with 100% response rates. Of these, 253(59.9%) were chemistry tests. From the expected < 90min TAT clinical chemistry tests, only 41(16.2%) and from < 60min TAT time for hematology tests, 37(21.9%) met the target. The laboratory TAT was affected by factors including high work load, laboratory information system problem, power interruption and sample collection time. Moreover, the level of knowledge, attitude and practices of laboratory staffs towards laboratory TAT were 60%, 85.7% and 62.9% respectively.

**Conclusion:**

Overall achievement of clinical Chemistry and hematology tests TAT was poor. The finding might reflect other public hospital situation in Addis Ababa. Thus, additional large scale studies need to conduct.

## Introduction

Medical Laboratory Turnaround Time (TAT) is one of the key quality indicators to measure performance of the laboratory, can be defined differently according to the test type (stat versus routine), analytic, and institution. It is usually defined as the time from when a test is ordered until the result is reported (1). On the other hand, many laboratory TAT is defined as the interval between “the time of sample collection and the laboratory report is arrived to the clinicians”, which is commonly known as diagnostic turnaround time (2,3). TAT is subdivided into pre-testing, testing and post-test. In the “total testing cycle”, TAT is described as grouping of different steps including barcode printing, sample collection, transportation, preparation, analysis, verification and reporting. Laboratory often gives importance on accuracy and precision of the tests as their goals for quality service (3).

In clinical laboratory activities, quality test result plays a significant role on medical diagnosis, decision, monitoring prognosis of disease with or without treatment. Studies indicated that up to 80% of medical decisions rely on laboratory test results (4). Thus, timely delivering of laboratory results is the crucial mainstays of a competent clinical laboratory, whose goal is to deliver a better-quality service to its clients. This attribute can be monitored very efficiently by establishing a test parameter, TAT (5).

Poor laboratory performance in terms of test TAT has a major impact on patient care, delayed test report, and TAT outliers, would have a major impact on the efficiency of diagnosis and management of patients (6).

The incidence of laboratory errors varies greatly depending on the steps of the total testing process (TTP). In particular, laboratory professionals should investigate and give attention to the pre-analytical and post-analytical phases, which have been demonstrated to be more susceptible to errors than the analytical phase. As studies indicated, the pre-analytical phase has the highest error rates, accounting for up to 70% of all mistakes in laboratory diagnostics (7).

As stated by the ISO 15189:2012, “in consultation with its users based on setting, staffing, workload, equipment, material and supplies, the laboratory shall establish appropriate turnaround times for each of its test to determine whether or not it is meeting the established target with regular assessment of the laboratory quality result with TAT” (8).

The total laboratory results TAT is influenced due to incompetency of phlebotomy, high workloads, inappropriate workflow, machine breakdown, delay in the maintenance of analyzers and computer shutdown. In addition, most laboratories do not stress enough the significance of TAT and give more importance to the accuracy of results. In summary, poor specimen transportation, shortage of resources, lack of knowledge and skills, power supply interruption, poor infrastructure and shortage of supplies are major factors that affect condition of quality laboratory services and TAT (9, 10).

Therefore, during assessment of the laboratory services, evaluation of established TAT for each test is an important tool (11,12). Ethiopian standards for comprehensive specialized hospital requirements (ES3618:2012), under section 5, states that each service unit of the hospital shall maintain a sufficient number of staff with the qualifications, training and skills necessary to meet patient needs. According to this standard each hospital should develop monitoring and evaluation tools to assess activities including, laboratory performance and workload. These tests are also the most significant rate-limiting step in patient management and discharge. In light of this background, in Armed Force Comprehensive Specialized Hospital, the status of TAT was not yet studied and documented. This study analyzed the gap of delaines from the sample collection to result release to physicians. This article is mainly focused on evaluation of performance of TAT for the clinical chemistry and hematology tests. In addition, the study identified the factors that affect the current turnaround time in the hospital's laboratory.

## Materials and Methods

**Study design and setting**: A hospital-based cross-sectional study was conducted at Armed Force Comprehensive and Specialized Hospital (AFCSH) laboratory, in the clinical chemistry and hematology departments, Addis Ababa, Ethiopia. The hospital is owned by the government under the Federal Ministry of Defense (FMoD); it is situated at western part of Addis Ababa, the capital city of Ethiopia. The hospital has 15 wards with five hundred ninety-four beds, which is a tertiary level super specialty hospital. It provides medical service to members of the defense force with their family, and civil servants in the institution. The hospital also provides services in its private wing for inpatient and ambulatory patients. It also functions as a referral center for different hospitals under the ministry of defense.

The hospital has facilities for laboratory services that help in diagnosis, treatment follow-ups and monitoring of diseases across different departments, including clinical chemistry and hematology departments. There are laboratory coordinators and vice coordinator, with 31 military and civil professionals having different levels and field of training, 13 phlebotomists and 3 runners. It is equipped with fully automated clinical chemistry analyzers, M-200E, M-200, coagulation analyzers and Mini Vidas for hormonal assay, fully automated hematology analyzers with Laboratory Information system, LIS. Clinical chemistry department performs a large number of tests: includes lipid profile, renal function, liver function, measurement of glucose and hormonal assays. On the other hand, hematology department performs different tests, including complete Blood Count, CBC, coagulation panels, blood morphology, blood film (BF) and erythrocyte sedimentation rates (ESR). According to the 2010 Ethiopian calendar annual reports of AFCSH, on average in the clinical chemistry and hematology departments, up 128 and 100 samples were run per day respectively (13). These tests were performed in clinical chemistry with three medical laboratory technologists [Bachelors of Science (BSc) degree holders in Medical Laboratory Sciences (MLS)], and one laboratory technician (Diploma holder) in MLS. On the other hand, the Hematology Laboratory had one Master's of Science degree holder in clinical laboratory sciences, hematology track and two BSc degree holders in MLS.

**Sample size determination and Sampling method**:

Single population proportion formula was used to determine the sample size with proportion study subjects = 50%, since there were no previous similar studies in Ethiopia. Considering 10% non response rate, the final sample size becomes 422.

The sample size considered those laboratory test requests comes into AFCSH laboratory both from outpatient and inpatients departments within the study periods. Thus, the samples were allocated proportionately for chemistry and hematology departments. These distributions of eligible test requests was done according to number of tests ordering between the two departments by using annual report in the laboratory (60% and 40% respectively). Systematic sampling technique was employed to collect the sampled test results. After identifying the random start by lottery method, data was collected in every five samples. In addition, all laboratory professionals and phlebotomists were participated in KAP assessment on TAT since the number of staff was small in number.

**Data collection and measurements of variables**: The data was collected by 3 trained data collectors. The data collections procedure was in short as follows: each sample TAT was assessed at three phases- pre-analytical, analytical and post-analytical phases. The three trained data collectors assigned at each phase followed the sample by exchanging information. At first, according to the selection criteria seated, where among the blood samples arrived in the laboratory, the fifth one from the previously chosen sample was selected. Then, sample bar code, date, the type of test (clinical chemistry, or hematology), and time of arrival for the pre-analytical processes were recorded. Then, when the samples moved to the analytical and post-analytical steps, TAT was assessed by respective data collectors following the data collection questionnaire sheets. Finally, the three data collectors compiled the recorded data sheets at each phase and kept them in locker box, for further data analysis.

On the other hand, structured questionnaire and on-site observational study was employed to collect data related with knowledge, attitude and practices of laboratory professionals and phlebotomist regarding about of TAT. Measurements of variables were conducted as per the following laboratory sated standards:

- TAT less and equal to 90 minutes is termed as good performance, and TAT greater than 90 minutes is termed as poor performance of the laboratory for clinical chemistry (14).
- TAT less and equal to 60 minutes is termed as good performance, and TAT greater than 60 minutes is termed as poor performance of the laboratory for hematology (14).
- Target TAT for chemistry and hematology: pre-analytical phases less and equal to 40 minutes and 30 minutes, for analytical phases less and equal to 20 minutes and 10 minutes for post-analytical phases less and equal to 30min and 20min respectively (14).
- Good knowledge: participants who answer the provided 10 questions to measure knowledge score and correct 6 and above
- Good attitude: participants who answer the provided 10 questions to measure attitude score and correct 6 and above
- Good practices: participants who answer the provided 10 questions to measure practices score and correct 6 and above

**Data quality assurance, data analysis and interpretation**: Prior to the actual work, training was given to data collectors by principal investigators, and the questionnaire was pre-tested in Air Force Hospital at Bishoftu, Ethiopia, to assure that it is proper and understandable. The collected data was checked daily for reliability. Each activity was controlled to ensure data quality. Data generated was entered and analyzed by Statistical Package for Social Sciences (SPSS) software version 24.0 (IBM, USA). Descriptive statistics was performed. Binary and multiple logistic regression analyses were done to find out statistically significant association and strength of association between dependent and independent variables at p-value <0.05 and COR and AOR with 95%CI.

**Operational definitions**: Four terms are defined below:

- TAT is defined as the interval between “the time of sample collection” and “the report dispatch to the physicians” (15).
- Scheduled test refers to clinical chemistry and hematology test orders for patients.
- Unscheduled test refers to clinical chemistry and hematology test orders for medical checkup.
- High patient flow refers to the number of patient flow, more than one hundred per day

**Ethical considerations**: The study was conducted after ethical approval was obtained from Department of Medical Laboratory Science Research and Ethics Review Committee, College of health sciences, Addis Ababa University. Written permission was obtained from the Armed Force Comprehensive Specialize Hospital (AFCSH) administrator. Participants were recruited after they were informed about the objectives and use of the study and after they gave informed consent. Samples were coded and confidentiality of patient data was maintained throughout the study.

## Results

**Figure 1:**
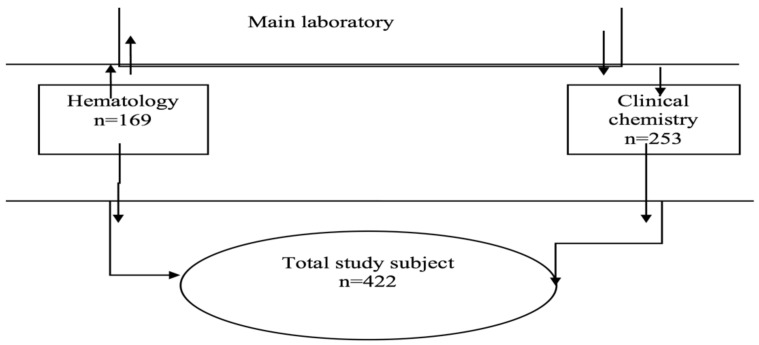
Proportional sample size allocations AFCSH laboratory staffs at the clinical chemistry and Hematology departments to assess TAT; Addis Ababa, Ethiopia, 2019.

**Frequency distribution of TAT for clinical chemistry and hematology test result**: A total of 422 laboratory test results were systematically selected with 100% completeness rate. Of the total, 253(59.9%) were chemistry while 169(40.1%) were hematology tests. From the expected below 90 minutes TAT set for clinical chemistry tests, only one sixth achieved the target time, 41/253(16.2%), whereas from the established less than 60 minutes TAT for hematology test, only one fourth, 37/169 (21.9%), met the target as shown in Figure 1. In our findings average delayed TAT in clinical chemistry and hematology tests were 184.96±74.928 minutes, and 139.85±88.118 minutes respectively.

Different challenges were faced during clinical chemistry and hematology test performances to achieve targeted TAT. Among them, the main problems identified both in clinical chemistry and hematology test were daily work load [(188/253 (74.3%) and 96/169 (56.8%) respectively,], and interrupted computer system [214/253 (84.6%), and 101/169 (59.8%) respectively)], mentioned ones (Table 1).

**Table 1:** Frequency distribution of TAT for chemistry test result and variables which may influence the achievement of TAT standard in AFCSH, Addis Ababa, 2019 (n=253)

Variables		Chemistry test sample	Hematology test sample
		N(%)	N (%)
IQC	Passed	246 (97.2%)	169(100%)
	Failed	7(2.8%)	0(0.0%)
	Total	253(100%)	169(100%)
Daily work load	Usual	65(25.7%)	73(43.2%)
	High	188(74.3%)	96(56.8%)
Computer system related	Uninterrupted	39(15.4%)	68(40.2%)
	Interrupted	214(84.6%)	101(59.8.%)
Test order	For Patient	115(45.5%)	84(49.7%)
	For screening	138(54.5%)	85(50.3%)
Power supply interruption	uninterrupted	181(71.5%)	144(85.2%)
	Interrupted	72(11%`)	25(14.8%)
Time of collection	8:00 – 9:59hr	204(80.4%)	117(69.2%)
	10:00 – 12:00hr	49(19.6%)	52(30.%)

Detailed analysis on difficulties to achieved targeted TAT in relation to daily laboratory work activity at the pre-analytical, analytical, and post-analytical period in both clinical chemistry and hematology tests was done. Accordingly, during pre-analytical phase, the majority TAT was not achieved both in clinical chemistry and hematology tests, 189/263 (74.7%) and 83/169 (49.1%) respectively. During analytical phase, the majority clinical chemistry tests achieved target TAT, 171/263 (67.6 %), whereas in hematology the majority tests did not achieve TAT, 54/169 (31.9%). During post-analytical phase, in both clinical chemistry and hematology tests, the majority TAT was not achieved, 141/263 (55.7%) and 82/169 (48.5%), respectively (Table 2).

**Table 2:** Correlation between pre-analytical, during analytical, post analytical with daily work load, (n= 253) of TAT standard in AFCSH, Addis Ababa, 2019

Variables		Clinical chemistry			Hematology		
	Daily work load	Achieved	Not Achieved	P-value	Achieved	Not Achieved	P-value
TAT for Pre analytical	Normal	10(3.9%)	24(9.5%)	0.019	49(28.9%)	24(14.2%)	<0.01
	High	30(11.6%)	189(74.7%)		13(7.7%)	83(49.1%)	
TAT for analytical	Normal	171(67.6%)	10(3.9%)	<0.01	49(28.9%)	24(14.2%)	0.03
	High	48(18.9%)	24(9.5%)		42(24.9%)	54(31.9%)	
TAT for post analytical	Normal	24(9.5%)	10(3.9%)	<0.01	37(21.9%)	36(21.3%)	<0.01
	High	78(30.8%)	141(55.7%)		14(8.3%)	82(48.5%)	

**Factors associated with delay in TAT of chemistry and hematology test**: To identify the associated factors for not achieving standard TAT for chemistry, both bivariate and multivariable logistic analyses were done on different selected variables. The bivariate analysis result showed that four variables (daily workload at the pre- and post-analytical stages, interrupted LIS, and type of test order) were statistically significantly associated with level of TAT performance (Table 3).

**Table 3:** Bivariate and multivariable logistic regression analysis of chemistry and Hematology tests, and factors associated with delay of TAT standard in AFCSH, Addis Ababa. 2019

variable		TAT for clinical chemistry					TAT for hematology				
		Not achieved (n=253)	COR (95%CI)	p-value	AOR (95%CI)	p-value	Not achieved (n=169)	COR (95%CI)	p-value	AOR (95%CI)	p-value
**work load at Pre-analytical**	Usual	24(70.4%)	1		1		24(32.9%)	1		1	
	High	189(86.3%)	2.62(1.14–6.03)	0.002	2.89(1.13–7.45)	0.006	83(86.5%)	13(6.9–22.92)	<0.001	8.98(2.01–21.47)	<0.001
**work load at** **Analytical**	Usual	10(5.5%)	1		1		24(32.9%)	1		1	10(5.5%)
	High	24(33.7%)	8.55(3.83–19.11)	<0.001	1.03(0.14–10.28)	0.995	54(56.3%)	2.62(1.39–4.94)	0.985	0.929(0.005–1.026)	24(33.7%)
**work load at Post-analytical**	Usual	10(29.4%)	1		1		31 (42.5%)	1		1	10(29.4%)
	High	141(62.4%)	4.39(1.97–9.54)	<0.001	1.76(4.78–12.45)	0.024	85 (88.5%)	6.02(2.90–12.48)	<0.001	5.14(1.26–19.68)	141(62.4%)
**LIS system**	Uninterrupted	15 (38.5%)	1		1		14 (28.0%)	1		1	15 (38.5%)
	Interrupted	197 (92.1%)	18.541 (8.22–41.82)	<0.001	3.19(1.14–8.92)	0.027	119 (100%)	14.57(5.82–36.49)	<0.001	6.21(1.57–24.53)	197 (92.1%)
**Test order**	For Patient	62 (63.9%)	1		1		53 (63.1%)	9.36(3.42–25.60)	<0.001	7.55(2.01–12.33)	62 (63.9%)
	For screening	150 (96.2%)	14.11 (5.65–35.24)	<0.001	6.02(2.190–16.52)	<0.001	80 (94.1%)	1		1	150 (96.2%)
**Power** **interruption**	Uninterrupted	143 (79.0%)	1		1		122 (77.2%)	1		1	143 (79.0%)
	Interrupted	69 (95.8%)	6.11 (1.82–20.49)	0.049	0.998(0.64–28.205)	0.962	11 (100%)	7.34(1.68–32.07)	0.008	3.03(0.46–23.27)	69 (95.8%)
**Time of collection**	8:00 – 9:59am	174 (85.3%)	1		1		101 (86.3%)	1		1	
	10:00–12:00am	38 (77.6%)	0.59 (0.27–1.29)	0.294	0.899(0.001–0.994)	0.999	32 (61.50%)	3.94(1.83–8.50)	<0.001	3.01(0.87–10.36)	0.049

Accordingly, in clinical chemistry tests pre- and post-analytical steps during high workload, the poor performances were seen more than 2 times [2.89(1.13 – 7.45)] and more than one time [1.76(4.78–12.45)] than the normal time. Similarly, interruption of ILS system more than 3 times [3.19(1.14 – 8.92)], and tests done only for screening purposes more than 6 times [6.02(2.190– 16.52)] contributed to the occurrence of failed TAT performances. On the other hand, in hematology tests, pre-analytical steps during high workload had the poor performances seen more than 8 times [8.98(2.01 – 21.47)] than the normal time. Similarly, interruption of LIS system more than 6 times [6.21(1.57 – 24.53)], and tests done only for screening purposes more than 7 times [7.55(2.01–12.33)] contributed to the occurrence of failed TAT performances (Table 3).

**Assessment of Knowledge, Attitude and Practices (KAP)**: A total of 35 laboratory Department work forces were participated in KAP assessment towards TAT performance. They had different qualifications and skills under laboratory profession. Most of the participants (40.0%) were laboratory technologists with BSc degree, and 34.3% were phlebotomists. Regarding work experience, 34.3% were £ 5 years, 31.4% were 6–10 years, and 34.3% were more than 10 years of experience in laboratory practices (Table 4). Table 4 shows the level of knowledge, attitude, practices and some characteristics of laboratory department staff, in AFCSH, Addis Ababa, 2019. (n=35).

**Table 4:** Table showed the level of knowledge, attitude, practices and some characteristics of laboratory department staffs, in AFCSH, Addis Ababa, 2019. (n=35)

Characteristics		Frequeny(%)
**Level of** **knowledge**	Good	21(60%)
	Poor	14(40%)
**Level of attitude**	Good	30(85.7%)
	Poor	5(14.3%)
**Level of practices**	Good	22(62.9% )
	Poor	13 (37.1%)
**Qualification**	Masters	3(8.6%)
	Lab technologist	14(40.0%)
	Lab technician	6(17.1%)
	Phlebotomist	12(34.3%)
**Work experience**	≤ 5 years	12(34.3%)
	6 – 10 years	11(31.4% )
	> 10 years	12(34.3%)
**Sex**	Male	17(48.6%)
	Female	18(52.4%)

On the detailed KAPs analysis of the study participants, among laboratory technologists, 10(71.3%) had good knowledge. The level of knowledge on TAT relatively decreased from those who had Master's degree to phlebotomists, whereas good attitude was very high among those who had Master's degree and Diploma holder laboratory technicians than phlebotomists and laboratory technologists with first degree. Level of good practices was nearly similar through different levels of qualification. Distribution of good knowledge and good practice increase along with increasing work experience, whereas good attitude was 100% among those with work experiences between 6 to 10 years (Table 5).

**Table 5:** Table showed the percentage relationship between the level of KAP and some related characteristics of participants, AFCSH, Addis Ababa, Ethiopia 2019

Characteristics		Level of knowledge		Level of attitude		Level of practices	
		Good	Poor	Good	Poor	Good	Poor
Qualification	Masters	3(100%)	0(0.0%)	3(100%)	0(0.0%)	2(66.7%)	1(33.3%)
	Lab technologist	10(71.3%)	4(28.7%)	11(78.6%)	3(21.4%)	9(64.3%)	5(35.7%)
	Lab technician	4(66.7%)	2(33.3%)	6(100%)	0(0.0%)	4(66.7%)	2(33.3%)
	Phlebotomist	6(50.0%)	6(50.0%)	10(83.3%)	2(16.7%)	7(58.3%)	5(41.7%)
Work experience	≤ 5 years	5(41.7%)	7(58.3%)	10(83.3%)	2(167%)	7(58.3%)	5(41.7%)
	6 – 10 years	8(72.7%)	3(27.3%)	11(100%)	0(0.0%)	7(63.6%)	4(36.4%)
	> 10 years	8(66.7%)	4(33.3%)	9(75.0%)	3(25.0%)	8(66.7%)	4(33.3%)
Sex	Male	12(70.6%)	5(29.4%)	16(94.1%)	1(5.9%)	12(70.6%)	5(29.4%)
	Female	9(50.0%)	9(50.0%)	14(77.8%)	4(22.2%)	10(55.6%)	8(44.4%)

## Discussion

Turnaround time is one of the laboratory performance indicators in the health service facility. The current study tried to measure the level of TAT in clinical chemistry and hematology tests in AFCSH, Addis Ababa, Ethiopia. This study showed that the performance of AFCSH laboratory in achievement of TAT in clinical chemistry and hematology sampled tests was considerably very low. The achievement of clinical chemistry tests was 41(16.3%) lower than the achievement of TAT reported in Korea (98.0%), India (59.3%) and Pakistan (54.6%), respectively (16, 17, and 18). In the present study, the average mean delay of clinical chemistry tests on TAT was very high, 184.96±74.928, and this was higher than that of the study conducted in the Republic of Korea, which was 43.6±7.7 (16), and the study done in Nepal, 54.6±8.4 minutes (19). The very significant different between the current study and others was due to high daily patient flow, type of laboratory machine, LIS interruption and the time of sample collection.

In our study, the level of hematology sampled tests achieved the target TAT was considerably very low, 37(21.6%). This is lower than the levels of TAT reported in Korea, and India, 79.0% and 54.6%, respectively, and also lower than the study done by Gungtok which was 62% (16,20,17). Besides, in the present study, the average dalliance TAT for hematology sampled tests were 139.85±88.118 minutes, whereas this delaines in a study conducted in Iran was 43.6±7.7 minutes (21) and in a study conduct in Canada was 90 minutes (22). Possible reasons for this difference were high number of daily patient flow, type of laboratory machine, and the time of data collection.

In the present study, one of the factors for delaines of TAT in clinical chemistry tests was high number of clients come to the laboratory in the morning between 8:00–9:59 hours. Our findings were comparable with a study conducted in India, where most of the delay in TAT of the tests occurred in the morning shift (17). Additionally, the test requested to chemistry laboratory test for medical screening purpose was significantly associated with delay of laboratory result from the recommended time, p-value <0.0001. Moreover, the medical laboratory size is another factor which affects the TAT of tests. It has been reported that laboratory results were available sooner in nonteaching than teaching and in smaller rather than larger institutions (17).

In the present study, the type of hematology test request between patient and medical screening showed significant differences on achieving targeted TAT. Hematology test analysis for patients seven times more likely create delaines to archive TAT than for those medical screening tests. The samples collected between 8:00 to 10:00am were three times more likely delayed in TAT than the samples collected after 10:00 to 12:00 am, during working of the day. Both conditions (test order and time of collection) had significant differences at p-value <0.0001, whereas studies conducted in Kenya and Iran revealed that daily workload and interruption in LIS significantly predicted the extended TAT of hematology test at a p-value <0.05 (21, 18).

Awareness of laboratory staff about TAT is one of the very important factors that influence achievement of overall TAT in defined time interval. The present study revealed that the level of average sums of knowledge, attitude and practices of AFCSH laboratory staff was 60%, 85.7% and 62.9%, respectively. In the current study, the poor awareness influences the level of turnaround time of clinical chemistry and hematology tests by 16.3% and 21.6%, respectively.

In addition, the overall average TAT was more delayed in our observational study. It may be due to laboratory instrumentation failure, shortage of data encoder in morning hours, accrual samples on sample collection site, not enough sample collectors in morning hours, and lack of exposure to the new LIS. In addition, shortage of deionizer water for chemistry machines, and un-interfaced the system with the automated laboratory analyzers could be reasons. In our study, the total laboratory TAT for chemistry and hematology tests was affected by those variables such as high workload, LIS and power interruption, unscheduled test order and sample collection time. The pre-analytical and post-analytical phases were also significantly affected by these two variables (LIS interruption and high work loud) at p-value <0.05.

Overall, the level of chemistry and hematology turnaround time as determined in the current study was very low, which is 16.2% for chemistry and 23.9% for hematology. Daily workload, unlimited flow of medical screening test, interruption of LIS, and pick time (8:00 – 9:59am) significantly influenced the achievement of recommended turnaround time of chemistry and hematology tests in the hospital. Daily workload had significant influence on pre-analytical phases of chemistry and hematology laboratory tests than on analysis and post analysis turnover time. Achieving the recommended TAT is crucial even to save the life of patients. So, based on the findings of the current study, the following corrective actions should be made:

- scheduled time for those clients who come to hospital for only medical fitness separately from the regular patients is required;
- The interruption of LIS should be managed to resolve its potential effect on the delay of result reporting;
- Assigning additional personnel/workforce in laboratory in the morning time when flow is picking is necessary.
- In light of our findings, further longitudinal studies can be planned to exactly define the grounds for such disparities in the findings.

## Figures and Tables

**Figure 2:**
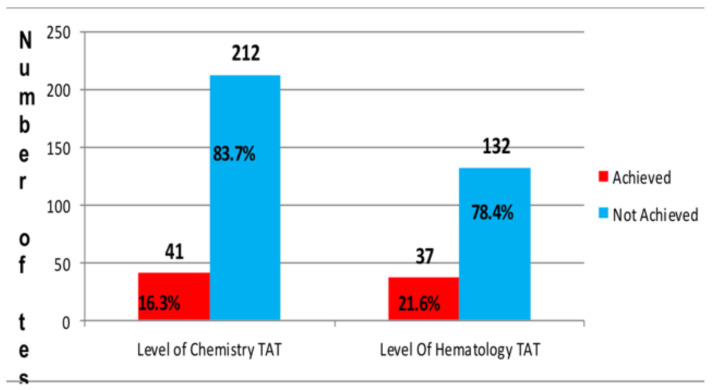
Level of Clinical chemistry and hematology test of turnaround time performances in AFCSH, Addis Ababa, Ethiopia, 2019.
